# Intraoperative dexmedetomidine on postoperative sleep disturbance in older patients undergoing major abdominal surgery: A randomized controlled trial protocol

**DOI:** 10.1016/j.heliyon.2024.e31668

**Published:** 2024-05-21

**Authors:** Xiu Yang, Jing-hui Hu, Li-ping Fan, Hui-ping Peng, Hai-jing Shi, Min-yuan Zhuang, Fu-hai Ji, Ke Peng

**Affiliations:** a Department of Anesthesiology, First Affiliated Hospital of Soochow University, Suzhou, Jiangsu, China; b Institute of Anesthesiology, Soochow University, Suzhou, Jiangsu, China; c Jintan Traditional Chinese Medicine Hospital, Changzhou, Jiangsu, China

**Keywords:** Postoperative sleep disturbance, Dexmedetomidine, Abdominal surgery, Sleep quality

## Abstract

**Background:**

Postoperative sleep disturbance (PSD) occurs frequently in patients who undergo major abdominal surgical procedures. Dexmedetomidine is a promising agent to improve the quality of sleep for surgical patients. We designed this trial to investigate the effects of two different doses of intraoperative dexmedetomidine on the occurrence of PSD in elderly patients who have major abdominal surgery.

**Methods:**

In this randomized, double-blind, controlled trial, 210 elderly patients aged ≥65 years will be randomized, with an allocation ratio of 1:1:1, to two dexmedetomidine groups (intraoperative infusion of 0.3 or 0.6 μg/kg/h) and a normal saline placebo group. The primary endpoint is the occurrence of PSD on the first night after surgery, assessed using the Athens Insomnia Scale. The secondary endpoints are (1) the incidence of PSD during the 2nd, 3rd, 5th, 7th, and 30th nights postoperatively; (2) pain at rest and on movement at 24 and 48 h postoperatively, assessed using the Numerical Rating Scale; (3) the incidence of postoperative delirium during 0–7 days postoperatively or until hospital discharge, assessed using the 3-min Confusion Assessment Method; (4) depressive symptoms during 0–7 days postoperatively or until hospital discharge, assessed using the 15-items Geriatric Depression Scale; and (5) quality of recovery on postoperative days 1, 2, and 3, assessed using the 15-items Quality of Recovery Scale. Patients' sleep data will also be collected by Xiaomi Mi Band 7 for further analysis.

**Discussion:**

The findings of this trial will provide clinical evidence for improving the quality of sleep among elderly patients undergoing major abdominal surgery.

**Ethics and dissemination:**

This trial was approved by the Ethics Committee of the First Affiliated Hospital of Soochow University (No. 2023-160). The results will be published in a peer-reviewed journal.

**Trial registration:**

Chinese Clinical Trial Registry (ChiCTR2300073163).

## 1 Introduction

Postoperative sleep disturbance (PSD), a common postoperative symptom among surgical patients, is characterized by sleep deprivation, sleep fragmentation and sleep architecture disturbances [[1], [2], [3]]. The reported incidence of PSD ranges from 15 % to 72 % and is especially high in older patients having major abdominal surgery [[4], [5], [6]]. Poor postoperative sleep results in many adverse outcomes, including neurocognitive disorders (such as delirium), pain perception alterations, inflammatory responses, metabolic derangements, and mood disturbances [[7], [8], [9], [10], [11], [12], [13]]. Hence, perioperative optimizing sleep quality in elderly surgical patients helps to reduce postoperative complications and healthcare costs.

PSD is associated with environment, surgical stress, anesthesia, and psychological factors [[14], [15], [16]]. Currently, approaches on improving quality of sleep in hospitalized adults mainly include noise controlling, psychological support, and medications (such as melatonin, benzodiazepines, and nonbenzodiazepine sedatives) [[17], [18], [19], [20], [21], [22]]. Dexmedetomidine (DEX), a selective α2-adrenergic receptor agonist possessing sedative, anxiolysis, sympatholytic, and analgesic effects, has been clinically used for sedation and anesthesia. DEX administration alters the arousal levels by reducing firing rate of locus coeruleus neurons and the release of norepinephrine [23]. Existing evidence shows that the use of DEX improved sleep quality for patients undergoing surgery or in intensive care unit [[24], [25], [26], [27]]. Nevertheless, whether DEX promotes a better sleep quality among older patients following abdominal surgery and its optimal dosage are unknown.

Therefore, we propose to conduct this randomized controlled trial assessing the effects of intraoperative DEX administration on the PSD occurrence in elderly patients having major abdominal surgical procedures. We will further compare DEX infusion at two different doses with normal saline placebo on the quality of sleep in our patients.

## 2 Methods

### 2.1 Study design

**Fig. 1:**
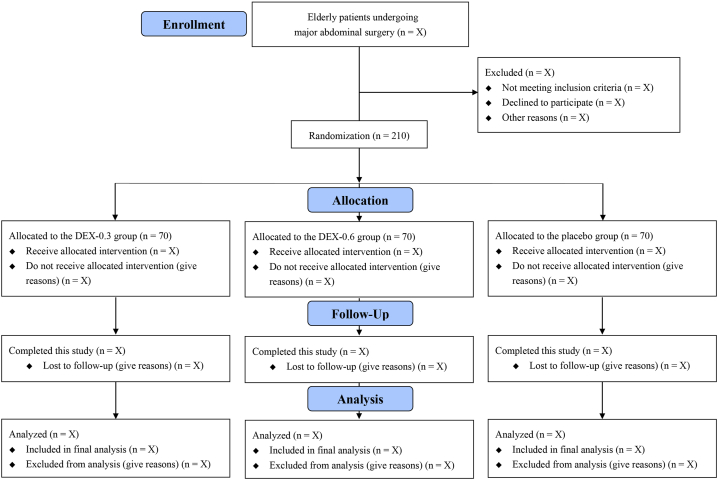
Flow chart of this trial.

In this randomized, double-blind, placebo-controlled, 3-arm, parallel-group trial, we will enroll 210 older patients undergoing major abdominal surgery at the First Affiliated Hospital of Soochow University, a tertiary teaching hospital in eastern China. Fig. 1 shows the trial flow diagram. After trial registration, the first subject was enrolled on August 1, 2023. We plan to complete patient enrollment by the end of 2024. This protocol adheres to the Standard Protocol Items: Recommendations for Interventional Trials (SPIRIT) guidelines (Supplemental file 1) [28]. The original Chinese version of this clinical trial protocol is available in Supplemental file 2.

### 2.2 Inclusion criteria

Patients will be included when they meet the following criteria:

(1) age ≥65 years;
(2) ASA physical status I–III;
(3) scheduled to undergo elective major abdominal surgery (both laparotomy and laparoscopic procedures with an estimated surgical duration ≥2 h) and be transferred to surgical wards postoperatively.

### 2.3 Exclusion criteria

The exclusion criteria are:

(1) sleep disorder (a preoperative Athens Insomnia Scale [AIS] score ≥6);
(2) severe bradycardia (heart rate <50 beats/min), sick sinus syndrome, second-degree or greater atrioventricular block without a pacemaker, cardiac insufficiency, hepatic or renal failure;
(3) long-term use of opioids, sedatives, antidepressants, or anxiolytics (defined by use for 90 days or longer);
(4) schizophrenia, Parkinson's disease, epilepsy, or myasthenia gravis;
(5) inability to communicate or refusal to participation.

### 2.4 Randomization and blinding

An independent researcher used the online tool (https://www.91trial.com) to generate the random numbers, with a 1:1:1 allocation ratio and block sizes of 3 and 6. The randomization results are stored in opaque sealed envelopes. Before anesthesia, a research nurse not involved in the randomization process opens the envelopes to assign patients into one of three study groups (DEX-0.3, DEX-0.6, and placebo) (Fig. 1). This nurse is also responsible for reconstituting the study medications into identical syringes labeled with the corresponding codes, which are then dispensed to attending anesthesiologists. Patients, surgeons, anesthesiologists, perioperative care providers, and postoperative observers will all remain masked to the details of assignment.

### 2.5 Anesthesia management

After entering the operating room, patients will be routinely monitored with electrocardiogram, peripheral saturation, and noninvasive blood pressure. Depth of anesthesia will be monitored using the bispectral index (BIS). Arterial blood pressure will be continuously monitored via a radial artery cannulation under local anesthesia with lidocaine. To avoid the potential impact of surgical time on circadian rhythms, all surgical procedures will start at 8:00–9:00 a.m. in the morning and are expected to finish before 1:00 p.m. Additionally, all patients will be managed according to a standardized protocol (maintaining a quiet and dim ward environment, provision of ear plugs and eye masks, and reducing interruptions from patient-care activities at night).

Anesthesia induction will be performed using propofol 1.5–2 mg/kg and sufentanil 0.3 μg/kg. After endotracheal intubation with cisatracurium 0.2 mg/kg, mechanical ventilation will be started to maintain the end-tidal carbon dioxide values within 35–45 mmHg. Anesthesia will be maintained using 1–3% sevoflurane inhalation adjusted to BIS values within 40–60, and remifentanil will be infused at a rate of 0.1–0.2 μg/kg/min. Additional cisatracurium will be given when needed. Patients will receive lactated Ringer's solution for fluid therapy, and colloids (gelatin or hydroxyethyl starch) can be used when indicated. Nasopharyngeal temperature at 36–37 °C will be achieved using a fluid warmer and/or a heating blanket. Dexamethasone and palonosetron will be used to prevent nausea and vomiting. Patients will be extubated in a post-anesthesia care unit (PACU). When an Aldrete score of ≥9 is reached, they can be discharged from the PACU to surgical wards. In the case of unplanned ICU admission, the patients will be excluded from the primary analysis.

For postoperative pain management, patients will be i.v. administered flurbiprofen axetil (50 mg) at the end of surgery, and receive patient-controlled analgesia (fentanyl 1 mg diluted with normal saline to a final volume of 100 mL) for 48 h postoperatively, with a background infusion of 1 mL/h, boluses of 2 mL, and a lockout time of 10 min. If significant pain still persists, patients could be given additional flurbiprofen axetil and fentanyl. Regional blocks or epidural analgesia will not be used in this study.

### 2.6 Study interventions

Patients in the two DEX groups will receive a continuous infusion of 0.3 μg/kg/h (in the DEX-0.3 group) or 0.6 μg/kg/h (in the DEX-0.6 group) from anesthesia induction until the beginning of skin closure. For patients in the placebo group, an equivalent volume of normal saline will be infused. These two infusion dosing regimens of DEX are based on a recent real-world cohort study in which intraoperative DEX at 0.2–0.8 μg/kg/h was associated with a decreased incidence of sleep disturbance after noncardiac major surgery [29].

### 2.7 Primary endpoint

The primary endpoint is the occurrence of PSD on the first night after surgery, assessed using the AIS by an independent observer who is unaware of group assignment via ward visits at 8–10 a.m. on the first postoperative day. The AIS is a widely used self-rated psychometric questionnaire comprising 8 items (i.e., sleep induction, waking after sleep onset, awakening, sleep duration, sleep quality, well-being, body function, and daytime sleepiness) [30]. The score in each item ranges from 0 to 3, and a lower score indicates a better sleep quality. The total AIS scores range from 0 to 24, and a score ≥6 indicates insomnia [30].

### 2.8 Secondary endpoints

The secondary endpoints are:

(1) the incidence of PSD during the 2nd, 3rd, 5th, 7th, and 30th nights postoperatively, assessed using the AIS via ward visits or by telephone calls after hospital discharge.
(2) pain intensity at rest and on movement at 24 and 48 h postoperatively, assessed using the Numerical Rating Scale (NRS). The total NRS scores range from 0 to 10, with 0 = painlessness and 10 = the worst pain.
(3) the incidence of postoperative delirium within 0–7 days after surgery or until hospital discharge (whichever occurs first), assessed using the 3-min Confusion Assessment Method (3D-CAM). The 3D-CAM comprises four features: (I) acute onset and mood swings, (II) inattention, (III) disorganized thinking, and (IV) altered consciousness. Patients who have both features I and II combined as either feature III or IV can be diagnosed as delirious [31,32].
(4) postoperative depressive symptoms within 0–7 days after surgery or until hospital discharge (whichever occurs first), assessed using the 15-items Geriatric Depression Scale (GDS-15). The GDS-15 is used to assess depressive symptoms in three dimensions (i.e., negative affect, cognitive distress, and behavioral inactivity), with a total score of 0–15 and a higher score indicating more serious depressive symptoms [33].
(5) the quality of recovery on postoperative days 1, 2, and 3, which will be evaluated with the use of the 15-items Quality of Recovery Scale (QoR-15). The QoR-15 is a patient-centered questionnaire assessing postoperative recovery quality with 15 items from five domains (i.e., physical independence, physical comfort, pain, psychological support, and emotional state). Each item has a 10-point numerical rating from 0 (a bad condition) to 10 (a good condition), and a higher total score represents a better quality of recovery [34,35].

### 2.9 Exploratory endpoints

The exploratory outcomes are patients' sleep data collected by Xiaomi Mi Band 7 (Xiaomi Inc) including total sleep time, sleep periods, light and deep sleep, rapid eye movement sleep, wake after sleep onset, and awake duration [36].

### 2.10 Data collection and monitoring

The schedule of this study is shown in Table 1. On the day before surgery, a well-trained investigator will screen the patients based on the eligibility criteria, collect baseline characteristic data (e.g., age, sex, BMI, ASA physical status, and comorbidities), and complete the preoperative assessment of sleep quality and depressive symptoms. Intraoperative data (e.g., vital signs, dose of anesthetics, surgical characteristics, and duration of surgery) will be collected in the electronic anesthesia system. The independent postoperative observer will conduct a follow-up of patients and complete the outcome assessments.

**Table 1:** Schedule of patient enrollment, study interventions, and outcome assessment.

Time point	Study period									
	Enrollment	Allocation	Post-allocation						Close-out	Follow-up
	*Preoperative visit*	1 h before surgery	Intra-operatively	Day1	Day2	Day3	Day5	Day7	*Hospital discharge*	30 days
**Patient enrollment**										
Eligibility criteria	×									
Written informed consent	×									
Demographic data	×									
Baseline characteristics	×									
Randomization/allocation		×								
**Study interventions**										
Dexmedetomidine-0.3			×							
Dexmedetomidine-0.6			×							
Placebo			×							
**Outcome assessment**										
Sleep quality (AIS)	×			×	×	×	×	×		×
Depressive symptoms (GDS)	×			×	×	×	×	×	×	
Delirium (3D-CAM)				×	×	×	×	×	×	
Recovery quality (QoR-15)				×	×	×				
Pain at rest (NRS)				×	×					
Pain on movement (NRS)				×	×					
Length of hospital stay									×	
Sleep data^a^				×	×	×				

According to SPIRIT statement of defining standard protocol items for clinical trials.

AIS, Athens Insomnia Scale; GDS, Geriatric Depression Scale; 3D-CAM, 3-min Confusion Assessment Method; QoR-15, Quality of Recovery-15; NRS, 1 Numeric Rating Scale.

a Exploratory data collected by Xiaomi Mi Band 7.

All data will be recorded in the case report forms (Supplemental file 3), which will be entered into an electronic database. Data collection is supervised by the principal investigator and an institutional independent data monitoring committee. The independent statistician will conduct statistical analyses using the datasets without patients' personally identifiable information.

### 2.11 Sample size

The previous studies showed that the prevalence of PSD in patients undergoing noncardiac major surgery ranged from 19 % to 44 %, and the administration of DEX was associated with a significantly decreased incidence [5,29,37,38]. In this trial, we hypothesize that the incidence of PSD is 25 %, 12 %, and 6 % in the placebo, DEX-0.3, and DEX-0.6 groups, respectively. Based on this assumption with a power of 80 % at a two-sided α level of 0.05, 189 patients are required to test the differences among the three groups. We assume a 10 % potential dropout rate, so we finally decide to enroll a total of 210 patients (n = 70 in each group). We estimated this sample size by using the software PASS (version 11.0; NCSS, LLC. Kaysville, Utah, USA).

### 2.12 Statistical analysis

The Kolmogorov-Smirnov test will be used for testing normal distribution. Continuous variables will be presented as means (standard deviations) if they are normally distributed; otherwise, they will be presented as medians (interquartile ranges). Categorical variables will be presented as numbers (percentages). To analyze data among three groups with correction for multiple comparisons, we will conduct one-way analysis of variance followed by the Dunnett method, Kruskal-Wallis test followed by the Dunn's method, and χ^2^ test followed by the Bonferroni method, as appropriate. For the primary and secondary endpoints, the treatment effects will be further analyzed using the relative risk or the difference in means or medians with 95 % confidence intervals.

The pre-specified exploratory analyses include (1) the overall effects of DEX (including patients in both DEX groups) vs. placebo, which will be analyzed using the unpaired *t*-test, Mann-Whitney rank-sum test, χ^2^ test, or Fisher's exact test, as appropriate; (2) the correlations between PSD and other outcome measures (e.g. pain intensity, delirium, depressive symptoms, and quality of recovery) using the Spearman analysis; and (3) subgroup analyses of PSD on the first postoperative night in terms of sex (male vs. female), preoperative AIS scores (0–3 vs. 4–6), and preoperative GDS-15 scores (0–5 vs. 6–15).

All analyses will be conducted in the modified intention-to-treat principle including all patients who undergo randomization and have available outcome data. Missing data will not be imputed. Statistical analysis will be done with SPSS software (version 25.0, SPSS Inc, Chicago, IL, USA). A two-sided *P* value less than 0.05 denotes a statistical significance. The original Chinese version of the statistical analysis plan is available in Supplemental file 2.

## 3 Discussion

In this study, we will investigate the effects of intraoperative infusion of DEX at two different doses on the quality of sleep among older patients who are planning to undergo major abdominal surgery. We will also evaluate the outcomes of postoperative pain intensity, delirium, depressive symptoms, and recovery quality after these surgical procedures. We will exploratorily observe the overall effects of DEX, the correlations between PSD and other outcomes, and the effects of DEX on PSD in several prespecified subgroups. Moreover, patients' sleep data from Xiaomi Mi Band 7 will be analyzed.

There are several perioperative risk factors of PSD for elderly patients undergoing surgical procedures. First, aging reduces the adaptability to new environment and is associated with a lower quality of sleep [39]. Second, patients with preoperative comorbidities such as obstructive sleep apnea, coronary artery disease and sleep disorders often have a bad quality of sleep [40,41]. Third, emotional problems, especially anxiety and depression, are closely related to PSD [42,43]. Next, the severity of surgical stress is also positively correlated with the occurrence of PSD [6]. Thus, elderly patients undergoing major abdominal surgical procedures are at an increased risk of PSD.

PSD may worsen neurocognitive dysfunction, impair the psychological state, and delay the recovery after surgery among elderly patients, leading to heavy emotional and financial burdens [44]. Substantial evidence shows that improving sleep quality could minimize the risk of postoperative delirium and enhance the quality of recovery in elderly patients [45,46]. However, non-pharmacological interventions such as earplugs and eye masks were ineffective to improve sleep quality after major abdominal surgery [47]. For these surgical patients, DEX maybe a promising candidate for PSD prevention, because DEX induces a state of unconsciousness parallel to natural sleep through activating the endogenous sleep-promoting pathways [48]. Moreover, the use of DEX may relieve sleep disorders owing to its anxiolytic, analgesic, and sleep-modulating effects [49]. A real-world retrospective cohort study suggested that intraoperative DEX infusion may reduce severe sleep disturbance after noncardiac major surgery [29]. A recent randomized trial showed that DEX administration during intracerebral surgery decreased postoperative delirium and improved the quality of sleep [50]. Intraoperative DEX may also promote the recovery of gastrointestinal function in older patients following abdominal surgery [51]. Therefore, we expect that intraoperative administration of DEX in our study exerts a prophylactic effect on PSD in the older adults undergoing major abdominal surgery.

Polysomnography (PSG) is regarded as the gold standard for diagnosis of sleep disorders in clinical settings and for sleep research. However, the high expenses of PSG and need for specialized staff make it difficult for application in many studies [52,53]. Recently, the wearable sleep monitors have been of high interest to researchers. Previous studies compared different wearable devices with PSG, suggesting that the common wearable devices had a limited specificity but could detect the basic sleep parameters with good accuracy and sensitivity [54,55]. Concheiro-Moscoso et al. reported that the Xiaomi Mi Band 5 could be used to record sleep data with 78 % accuracy and 89 % sensitivity [36]. In our study, Xiaomi Mi Band 7 will be used to collect patients' sleep parameters for further analysis on the clinical implication of DEX.

There are some limitations that need to be acknowledged. First, this is a single-center trial and our patients may not be representative of patients in other medical institutions. Second, we mainly focus on the primary outcome of PSD, and the current sample size may be insufficient to detect differences in the secondary outcomes. Last, detailed information on sleep is not acquired by the PSG or electroencephalography in our patients; however, we will use the Xiaomi Mi Band 7 to collect sleep data for exploratory analysis.

In summary, our trial will assess the effects of intraoperative DEX infusion at two different doses on the occurrence of PSD in elderly patients who undergo major abdominal surgery. Our results will provide evidence for improving the quality of sleep in these surgical patients.

## 4 Ethics and dissemination

This trial protocol was reviewed and approved by the Ethics Committee of the First Affiliated Hospital of Soochow University (No. 2023-160) on June 27, 2023 (Supplemental file 4). After that, the protocol was registered at the Chinese Clinical Trial Registry (identifier: ChiCTR2300073163) on July 3, 2023. Available at: https://www.chictr.org.cn/showproj.html?proj=199410. The study will be performed following the Declaration of Helsinki. All subjects will give their written informed consent to participate in this trial (Supplemental file 5). The study results will be submitted and published in a peer-reviewed journal.

## Funding

This work is supported by the Suzhou Key Laboratory of Anesthesiology (SZS2023013), Key Medical Research Projects in Jiangsu Province (ZD2022021), Suzhou Clinical Medical Center for Anesthesiology (Szlcyxzxj202102), Suzhou Medical Health Science and Technology Innovation Project (SKY2022136), 10.13039/501100004608Natural Science Foundation of Jiangsu Province (BK20230214) and Suzhou Talents (GSWS2023023). The funders have no role in the study design and are not involved in the preparation or submission of the manuscript for publication.

## Patient and public involvement

Patients and the public will not be involved in the design, recruitment, conduct, or report of the study. The study results will be disseminated to the participants via telephone or email.

## Data availability statement

No data was used for the research described in the article.

## CRediT authorship contribution statement

**Xiu Yang:** Writing – original draft, Project administration, Methodology, Investigation, Funding acquisition, Formal analysis, Data curation, Conceptualization. **Jing-hui Hu:** Writing – original draft, Project administration, Methodology, Investigation, Formal analysis, Data curation, Conceptualization. **Li-ping Fan:** Writing – original draft, Project administration, Methodology, Investigation, Formal analysis, Data curation, Conceptualization. **Hui-ping Peng:** Writing – original draft, Project administration, Methodology, Investigation, Conceptualization. **Hai-jing Shi:** Writing – original draft, Project administration, Methodology, Investigation. **Min-yuan Zhuang:** Writing – original draft, Project administration, Methodology, Investigation, Conceptualization. **Fu-hai Ji:** Writing – review & editing, Validation, Supervision, Funding acquisition, Formal analysis, Data curation, Conceptualization. **Ke Peng:** Writing – review & editing, Visualization, Validation, Supervision, Methodology, Funding acquisition, Formal analysis, Data curation, Conceptualization.

## Declaration of competing interest

The authors declare that they have no known competing financial interests or personal relationships that could have appeared to influence the work reported in this paper.
