# Supplementary material for: Intraoperative dexmedetomidine on postoperative sleep disturbance in older patients undergoing major abdominal surgery: A randomized controlled trial protocol
Source: Heliyon. 2024 May 21;10(11):e31668. doi: 10.1016/j.heliyon.2024.e31668 (PMC11153091; doi:10.1016/j.heliyon.2024.e31668)
Supplement: Multimedia component 2 [file mmc2.pdf]

研究方案

版本号：1.0

版本日期： 2023 年 06 月 07 日

一、项目名称

右美托咪定对腹部大手术老年患者术后睡眠质量的影响：一项随机对照研究

二、研究背景

术后睡眠障碍 (Postoperative sleep disturbance，PSD)作为术后常见症状之一，表现为睡眠剥夺、睡眠节律紊乱、睡眠体系异常、快慢波睡眠比例改变等，发生率约 15~72%[1,2]。PSD 可导致认知功能下降、术后疼痛加剧、术后疲劳综合征及心血管意外事件的发生，最终影响术后恢复质量，减缓恢复速度[3,4]。

PSD 是多种因素共同作用的结果，包括手术炎症反应、疼痛、麻醉药物的使用、术后恶心呕吐以及对病房环境的不适应等[5]。目前主要从"物理、心理、药物"三方面改善术后睡眠质量，如通过控制环境噪声和光照水平来缓解手术后严重的睡眠障碍，佩戴眼罩或耳塞，加强医患沟通，使用短效非苯二氮卓类药物、褪黑素和采取多模式镇痛等。然而，目前较少研究在手术过程中进行早期干预以降低手术后睡眠障碍的发生。

右美托咪定（Dexmedetomidine, DEX）是一种 α2 肾上腺素受体激动剂，常作为麻醉辅助用药，起到镇静、抗焦虑、镇痛和抑制交感神经等作用。当前研究发现，DEX 有改善老年术后患者睡眠质量的潜力[6,7]。这可能是因为 DEX 作用于桥脑蓝斑区，抑制蓝斑来源的去肾上腺素能神经传递到腹外侧视前核，而蓝斑核是接收外部刺激和唤醒睡眠的部位[8]。但目前尚无研究明确不同剂量的 DEX 对术后睡眠质量的改善程度是否有区别[9]。

因此，本课题拟开展一项前瞻性随机对照的临床研究，通过术中输注不同剂量的右美托咪定或安慰剂，评估对于腹部大手术老年患者术后 PSD 发生率、术后恢复质量、疼痛等情况的影响，为腹部大手术老年患者围术期麻醉用药管理提供实验依据，促进围术期恢复质量的改善。

三、研究目的

评估术中输注右美托咪定对于腹部大手术老年患者术后睡眠质量的影响，以及不同剂量

是否存在区别。指导麻醉管理，提高患者康复质量。

四、研究设计（包括研究的总体设计、样本量、参研单位数量、研究步骤和研究时限等）

1、总体设计

本研究是一个由研究者发起的，单中心、随机、双盲、对照研究。

2、样本量计算

参考既往研究，我们发现非心脏大手术患者术后睡眠障碍发生率约 19.6~44%，使用 DEX 可使发生率下降 50%，而高剂量 DEX 的发生率则更低，我们假设 PSD 发生率为 25%，低剂量 DEX 使发生率变为 12%，高剂量 DEX 使发生率变为 6%，使用 PASS 11 软件（NCSS, LLC. Kaysville, Utah, USA）进行样本量计算，采用双侧检验 α 为 0.05，效应量为 80%，共需 189 例样本量，考虑 10%的失访率，计划纳入 210 例患者，各组为 70 例。

3、参研单位

苏州大学附属第一医院

4、伦理和注册

获得伦理申请审批后，将在 WHO 一级注册机构中国临床试验注册中心进行在线注册并取得临床试验注册号。临床试验注册在入组第一例患者前完成。所有入组的患者均充分告知本研究的过程，并签署书面知情同意书。

5、 研究步骤

术前一天访视病人，签署麻醉及科研项目的知情同意书，向病人说明相关评分量表的使用方法并进行基本资料收集（年龄、性别、受教育程度、BMI、合并症）及术前睡眠、焦虑情况。手术当天不用术前用药，术前禁食 6 小时。患者入手术室后的标准监测包括：心电图（ECG），脉搏氧饱和度（SpO2），无创血压（NIBP），BIS 监测麻醉深度。完成外周静脉穿刺置管，并开始基础液体输注（平衡液，5 ml/kg/h）。常规麻醉诱导后行气管插管，连接呼吸机控制呼吸，吸入氧浓度 50%，容量控制模式，潮气量 8 ml/kg，频率 12-15 次/分，吸呼比 1:2，控制呼气末二氧化碳 PetCO2 在 35–45 mmHg。采用静吸复合方式维持麻醉（七氟烷 1~2%，静脉泵注 0.05–0.1 μg/kg/min 瑞芬太尼，间断静脉注射舒芬太尼 0.1 μg/kg 及顺式阿曲库铵 0.1 mg/kg），根据脑电双频指数 BIS 监测数值，调节麻醉深度。

实验组分为高剂量 DEX 组及低剂量 DEX 组，诱导后术中以 0.3ug/kg/h（低剂量）或 0.6ug/kg/h（高剂量）泵注。对照组以同等剂量的生理盐水进行负荷量及维持量的泵注。泵注时间为外科医生切皮开始至缝皮结束。

术中麻醉医生给予患者输注乳酸林格氏液或胶体（明胶或羟乙基淀粉）进行容量治疗，若患者的血红蛋白低于 7g/L 或出血量大于自身血容量的 30%，需及时输注少白红细胞和新鲜冷冻血浆补充血容量。输注液体过程中需使用液体加温仪进行加温，术中维持患者鼻咽温度在 36 到 37℃。低血压定义为：平均动脉压（mean arterial pressure, MAP）降低至基线值的 30%；心动过缓定义为：心率（heart rate, HR）小于 45 次/分钟。麻醉医生需针对低血压和心动过缓事件进行干预，包括：输液、注射去氧肾上腺素、麻黄碱或/和阿托品。手术后，由麻醉医师护送患者至麻醉后恢复室（post anesthesia care unit, PACU）继续监护，等待麻醉苏醒并拔除气管导管。气管导管拔除后，麻醉护士对患者进行改良的 Aldrete 评分（包括：肌力、呼吸、循环、氧和、神志，每项 2 分，总分为 10 分），若 Aldrete 评分≥9 分，麻醉护士护送患者由 PACU 转至普通病房进行下一步治疗。

麻醉医生在诱导后给予地塞米松 5mg，术毕给予帕洛诺司琼 0.25mg 预防术后恶心呕吐。术后镇痛使用患者自控式静脉镇痛，镇痛输注泵内加入芬太尼 1.0 mg，使用 0.9%的生理盐水稀释至 100 ml，背景剂量速度设置为 1 ml/小时，自控剂量设置为 2 ml，锁时时间设置为 15 分钟。病区护士对手术患者进行每日数字疼痛量表（NRS）评分，若 NRS 评分≥4 分，则上报管床医生下达静脉注射芬太尼和/或氟比洛芬酯医嘱进行额外镇痛。

（1）术前量表评估

a.简易精神状态评价表（MMSE）：该量表总分为 30 分，测试内容包括：时间和地点回忆、单词重复、算数（100 连续减 7）、言语表达、言语理解、简单指令运动[10]。若 MMSE 评分小于 23 分则认为该患者存在术前认知障碍[11]。

b.衰弱量表（The FRAIL Scale）：该量表包括 5 个条目，从疲劳、抗阻力、有氧运动、自身疾病、身体丢失五个方面评估，各方面占 1 分。0 分代表健康，1~2 分考虑为衰弱前期，3~5 分考虑为衰弱综合征[12]。

c.简版老年抑郁量表（GDS-15）：该量表从负面情绪，认知困扰和消极的行为/社会活动三个方面对老年人群进行简单有效的抑郁症筛查，分数＞5 分提示可能抑郁，≥10 分表示抑郁，且越高表明抑郁症状的严重程度越高[13,14]。

d. 阿森斯失眠量表（AIS）：该量表包括 8 个项目，即入睡时间、夜间觉醒、早醒、总睡眠时间、总睡眠质量、日间情绪、日间身体功能和日间嗜睡。项目评分范围从 0（完全没有问题）到 3（非常严重的问题），总分为 0-24，分数越低表示睡眠质量越好，总分≥6 分反映睡眠障碍的诊断[15]。

（2）术后量表评估

a.术后 AIS 及 GDS-15 评估

b. 3 分钟意识模糊评估法（3-minute Confusion Assessment Method, 3D-CAM）： 3D-CAM 的特征包括：①急性起病和情绪波动；②注意力不集中；③思维混乱；④意识改变。当特征①和特征②同时出现，且存在特征③或特征④时，临床诊断存在术后谵妄 [16,17]。

c.恢复质量量表（QoR-15）：该量表每项采用 0~10 分的计分方法，“0”提示一直都不能达到该种情况（状态不好），“10”提示一直都能达到该种情况（状态很好）。15 项相加得出总分，满分 150 分，总分越高提示术后恢复越好[18,19]。

d.疼痛数字评估量表（NRS）：将一条直线平均分成 10 份，在每个点用数字 0～10 分表示疼痛依次加重的程度，0 分为无痛，10 分为剧痛。1~3 分提示轻度疼痛、4~6 分提示中度疼痛、7~10 分提示重度疼痛。

五、研究人群（包括纳入标准、排除标准、退出终止标准等）

1、纳入标准

1）年龄≥65 岁，性别不限；

2）美国麻醉医师协会（American society of Anesthesiologists, ASA）分级 I-III 级；

3）行择期腹部大手术，预计手术时间≥2 小时，术后转入病房；

4）清楚了解实验过程并自愿参加，签署知情同意书。

2、排除标准

1）术前睡眠障碍（AIS≥6）；

2）严重心功能不全，严重肝功能不全（Chil-Pugh C 级），肾功能衰竭；

3）长期使用阿片类药物、镇静剂、抗抑郁药或抗焦虑药物；

4）精神分裂症，癫痫，帕金森症或重症肌无力病史；

5）不能进行有效沟通或拒绝参与研究。

3、退出或终止标准

受试者撤回知情同意书；完成 1 月随访后研究终止。

六、干预措施

手术期间右美托咪定以 0.3 ug/kg/h 或 0.6 ug/kg/h 或生理盐水安慰剂以相同的速度泵注。泵注时间为外科医生切皮开始至缝皮结束。

七、观察指标

1）术前：评估并记录抑郁、睡眠、疼痛等评分，记录人口统计学特征（性别、年龄、身高、体重）。

2）术中：记录入室时患者的生命体征（HR、BP、SpO2），电脑自动采集术中生命体征数据；记录麻醉药物使用总量（丙泊酚、瑞芬太尼、舒芬太尼、右美托咪定）、术中容量治疗、手术时间、拔管时间、PACU。

3）术后：记录术后各量表评分、PCIA 内芬太尼使用量，记录术后住院天数，住院期间生存情况及并发症，第 30 天睡眠情况。

八、研究流程图

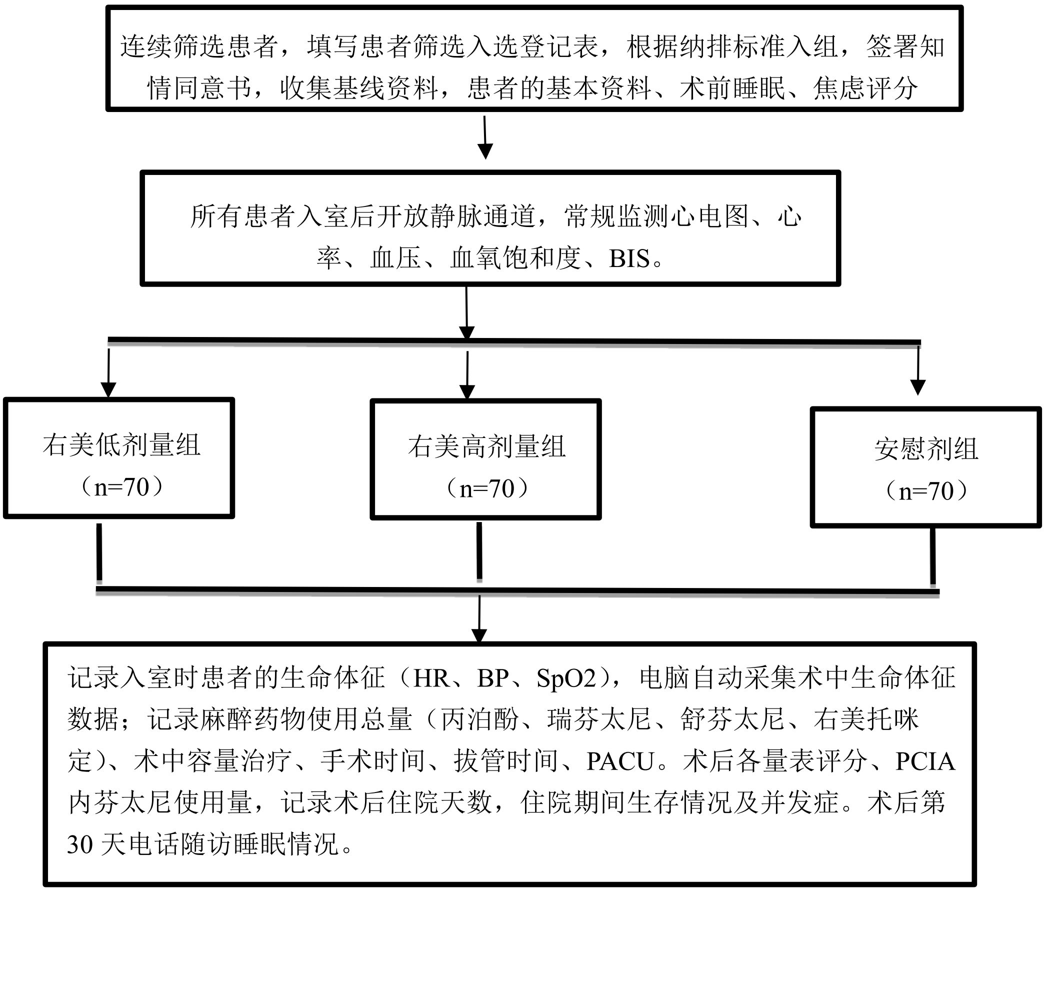

九、主要结局

本研究的主要结局指标为术后第 1 天 PSD 的发生率。

十、次要结局

本研究的次要结局为术后抑郁情况、术后谵妄发生率、术后恢复质量、术后24小时和48小时静息和活动时的NRS疼痛评分、术后30天内的AIS评分。

十一、随访计划

分别于术后1~7天进行病房随访，30天进行电话随访。随访内容为PSD、抑郁、疼痛、谵妄、术后并发症。

十二、研究的安全性监测、不良事件的处理方案

(1) 安全性监测

本研究中的干预措施为右美的静脉泵注均在常规麻醉管理可接受范围内，不会给受试者增加超出常规诊疗以外的风险，术中由上级主治及以上级别麻醉医生对整个麻醉过程进行安全性监测。

(2) 不良事件处理方案

术中如发生低血压（收缩压< 90 mmHg或MAP降低幅度超过基础值30%），则静脉注射麻黄素10 mg；如发生严重心动过缓（HR< 45次/min），则静脉注射阿托品0.5 mg；若发生高血压（收缩压>140mmHg或MAP升高幅度超过基础值30%）和心动过速（HR>100次/min），在麻醉深度和镇痛充分的情况下，可以静脉注射乌拉地尔5 mg或艾司洛尔20 mg。若术中或术后发生低氧血症（SpO_2＜90%），面罩加压辅助呼吸，行血气分析并对症处理。

十三、研究数据的管理与统计分析

Kolmogorov-Smirnov检验将用于检验正态分布。如果连续变量是正态分布的，则将其表示为均值（标准差）；否则，它们将显示为中位数（四分位间距）。类别变量将以数字（百分比）表示。为了分析三组之间的数据并对多重比较进行校正，我们将进行单向方差分析，然后采用Dunnett方法，Kruskal-Wallis检验，然后采用邓氏方法，以及χ^2检验，然后再采用Bonferroni方法（视情况而定）。对于主要和次要终点，将使用95%置信区间的相对风险或平均值或中位数的差异来进一步分析治疗效果。

预先规定的探索性分析包括（1）DEX（包括两组患者）与安慰剂的总体效果，将酌情使用不配对t检验、Mann-Whitney秩和检验、χ^2检验或Fisher精确检验进行分析；（2）使用Spearman分析的PSD与其他结果指标（如疼痛强度、谵妄、抑郁症状和恢复质量）之间的相关性；以及（3）术后第一晚PSD的性别（男性与女性）、术前AIS评分（0-3与4-6）

和术前 GDS-15 评分（0-5 与 6-15）的亚组分析。

所有分析都将按照修改后的意向治疗原则进行，包括所有接受随机化并有可用结果数据的患者。缺失的数据不会被估算。将使用 SPSS 软件（25.0 版，美国伊利诺伊州芝加哥 SPSS 公司）进行统计分析。双侧 P 值小于 0.05 表示具有统计学意义。

十四、参考文献

1.Chouchou F, Khoury S, Chauny JM, Denis R, Lavigne GJ. Postoperative sleep disruptions: a potential catalyst of acute pain? Sleep Med Rev. 2014 Jun;18(3):273-82.

2.隆巧玉,葛亚丽.麻醉管理对术后睡眠障碍的影响研究进展[J].大连医科大学学报,2022,44(03):244-248.

3.Duan G, Wang K, Peng T, Wu Z, Li H. The Effects of Intraoperative Dexmedetomidine Use and Its Different Dose on Postoperative Sleep Disturbance in Patients Who Have Undergone Non-Cardiac Major Surgery: A Real-World Cohort Study. Nat Sci Sleep. 2020 Mar 12;12:209-219.

4.Sui X, Wang Y, Jin M, Li K, Jiang G, Song A, He Z, Yin C, Zhao J, Wang L, Han F. The effects of dexmedetomidine for patient-controlled analgesia on postoperative sleep quality and gastrointestinal motility function after surgery: A prospective, randomized, double-blind, and controlled trial. Front Pharmacol. 2022 Oct 10;13:990358.

5.Su X, Wang DX. Improve postoperative sleep: what can we do? Curr Opin Anaesthesiol. 2018 Feb;31(1):83-88.

6.Song AH, Kucyi A, Napadow V, Brown EN, Loggia ML, Akeju O. Pharmacological Modulation of Noradrenergic Arousal Circuitry Disrupts Functional Connectivity of the Locus Ceruleus in Humans. J Neurosci. 2017 Jul 19;37(29):6938-6945.

7. Chen Z, Tang R, Zhang R, Jiang Y, Liu Y. Effects of dexmedetomidine administered for postoperative analgesia on sleep quality in patients undergoing abdominal hysterectomy. J Clin Anesth. 2017 Feb;36:118-122.

8. Wu XH, Cui F, Zhang C, Meng ZT, Wang DX, Ma J, Wang GF, Zhu SN, Ma D. Low-dose Dexmedetomidine Improves Sleep Quality Pattern in Elderly Patients after Noncardiac Surgery in the Intensive Care Unit: A Pilot Randomized Controlled Trial. Anesthesiology. 2016 Nov;125(5):979-991.

9. Liu H, Wei H, Qian S, Liu J, Xu W, Luo X, Fang J, Liu Q, Cai F. Effects of dexmedetomidine on postoperative sleep quality: a systematic review and meta-analysis of randomized controlled trials. BMC Anesthesiol. 2023 Mar 21;23(1):88. doi: 10.1186/s12871-023-02048-6. Erratum in: BMC Anesthesiol. 2023 Apr 3;23(1):111.
10. Jia X, Wang Z, Huang F, Su C, Du W, Jiang H, et al. A comparison of the Mini-Mental State Examination (MMSE) with the Montreal Cognitive Assessment (MoCA) for mild cognitive impairment screening in Chinese middle-aged and older population: a cross-sectional study. BMC Psychiatry. 2021;21(1):485.
11. Chi YL, Li ZS, Lin CS, Wang Q, Zhou YK. Evaluation of the postoperative cognitive dysfunction in elderly patients with general anesthesia. Eur Rev Med Pharmacol Sci. 2017;21(6):1346-54.
12. Abellan van Kan G, Rolland Y, Bergman H, et al. The I.A.N.A Task Force on frailty assessment of older people in clinical practice [J]. The Journal of Nutrition, Health & Aging, 2008, 12(1): 29-37.
13. Smith PJ, Attix DK, Weldon BC, Monk TG. Depressive Symptoms and Risk of Postoperative Delirium. Am J Geriatr Psychiatry. 2016 Mar;24(3):232-8.
14. Greene NH, Attix DK, Weldon BC, Smith PJ, McDonagh DL, Monk TG. Measures of executive function and depression identify patients at risk for postoperative delirium. Anesthesiology. 2009 Apr;110(4):788-95.
15. Song B, Li Y, Teng X, Li X, Yang Y, Zhu J. Comparison of morning and evening operation under general anesthesia on intraoperative anesthetic requirement, postoperative sleep quality, and pain: a randomized controlled trial. Nat Sci Sleep. 2020;12:467-475.
16. Marcantonio ER, Ngo LH, O'Connor M, Jones RN, Crane PK, Metzger ED, et al. 3D-CAM: derivation and validation of a 3-minute diagnostic interview for CAM-defined delirium: a cross-sectional diagnostic test study. Ann Intern Med. 2014;161(8):554-61.
17. Ely EW, Inouye SK, Bernard GR, Gordon S, Francis J, May L, et al. Delirium in mechanically ventilated patients: validity and reliability of the confusion assessment method for the intensive care unit (CAM-ICU). Jama. 2001;286(21):2703-10.
18. Wessels E, Perrie H, Scribante J, Jooma Z: Quality of recovery in the perioperative setting: A narrative review. J Clin Anesth 2022, 78:110685.

19. Kleif J, Gogenur I: Severity classification of the quality of recovery-15 score-An observational study. J Surg Res 2018, 225:101-107.
