# Supplementary material for: Intraoperative dexmedetomidine on postoperative sleep disturbance in older patients undergoing major abdominal surgery: A randomized controlled trial protocol
Source: Heliyon. 2024 May 21;10(11):e31668. doi: 10.1016/j.heliyon.2024.e31668 (PMC11153091; doi:10.1016/j.heliyon.2024.e31668)

Case report forms for PSD

<table>
<tr><td colspan="6">Basic information</td></tr>
<tr><td>Name</td><td></td><td colspan="3">Hospitalization ID</td><td></td></tr>
<tr><td>Age</td><td></td><td colspan="3">Height</td><td>cm</td></tr>
<tr><td>Weight</td><td>kg</td><td colspan="3">BMI</td><td>kg/cm^2</td></tr>
<tr><td colspan="2">Gender</td><td colspan="4">□Male □Female</td></tr>
<tr><td colspan="2">ASA score</td><td colspan="4">□I □II □III</td></tr>
<tr><td colspan="2">Smoking history</td><td colspan="4">□Never □Past □Current</td></tr>
<tr><td rowspan="5">Preoperative comorbidities</td><td colspan="5">□Hypertension</td></tr>
<tr><td colspan="5">□Cardiovascular system____</td></tr>
<tr><td colspan="5">□Cerebrovascular system____</td></tr>
<tr><td colspan="5">□Diabetes</td></tr>
<tr><td colspan="5">□Respiratory system____</td></tr>
<tr><td colspan="6">Preoperative scale score</td></tr>
<tr><td>GDS-15 score</td><td colspan="5"></td></tr>
<tr><td>AIS score</td><td colspan="5"></td></tr>
<tr><td colspan="6">Perioperative recording</td></tr>
<tr><td>Type of surgery</td><td colspan="5"></td></tr>
<tr><td>Duration of surgery</td><td colspan="5">____h____min</td></tr>
<tr><td>Total dose of DEX</td><td colspan="5"></td></tr>
<tr><td>Adverse events</td><td colspan="5">□Hypotension □Hypertension □Bradycardia
□Tachycardia □Hypoxemic □Other____</td></tr>
<tr><td colspan="6">PACU recording</td></tr>
<tr><td>Extubation time</td><td colspan="5"></td></tr>
<tr><td>Duration in PACU</td><td colspan="5">____h____min</td></tr>
<tr><td>Adverse events</td><td colspan="5">□Nausea and vomiting □Hypotension □Hypertension
□Tachycardia □Hypoxemic □Other____</td></tr>
<tr><td colspan="6">Postoperative recording</td></tr>
<tr><td colspan="3">Postoperative hospitalization duration</td><td colspan="3">____days</td></tr>
<tr><td>Postoperative Complication</td><td colspan="5"></td></tr>
<tr><td colspan="6">Postoperative scale score</td></tr>
<tr><td></td><td>Day 1</td><td>Day 2</td><td>Day 3</td><td>Day 5</td><td>Day 7</td></tr>
<tr><td>AIS score</td><td></td><td></td><td></td><td></td><td></td></tr>
<tr><td>3D-CAM</td><td></td><td></td><td></td><td></td><td></td></tr>
<tr><td>GDS-15 score</td><td></td><td></td><td></td><td></td><td></td></tr>
<tr><td>QoR-15 score</td><td></td><td></td><td></td><td>×</td><td>×</td></tr>
<tr><td>NRS score at rest</td><td></td><td></td><td></td><td>×</td><td>×</td></tr>
<tr><td>NRS score at movement</td><td></td><td></td><td></td><td>×</td><td>×</td></tr>
<tr><td colspan="6">Follow-up recording (Day 30)</td></tr>
<tr><td colspan="3">AIS score</td><td colspan="3"></td></tr>
</table>

Appendix 1. 15-items Geriatric Depression Scale (GDS-15)

Question / Item	Subscale	Score
Are you basically satisfied with your life?	Negative Affect	□Yes=0 □No=1
Have you dropped many of your activities and interests?	Behavioral Activation	□Yes=1 □No=0
Do you feel that your life is empty?	Cognitive Distress	□Yes=1 □No=0
Do you often get bored?	Behavioral Activation	□Yes=1 □No=0
Are you in good spirits most of the time?	Negative Affect	□Yes=0 □No=1
Are you afraid that something bad is going to happen to you?	-	□Yes=1 □No=0
Do you feel happy most of the time?	Negative Affect	□Yes=0 □No=1
Do you often feel helpless?	Negative Affect	□Yes=1 □No=0
Do you prefer to stay at home, rather than going out and doing new things?	Behavioral Activation	□Yes=1 □No=0
Do you feel you have more problems with memory than most people?	-	□Yes=1 □No=0
Do you think it is wonderful to be alive?	Negative Affect	□Yes=0 □No=1
Do you feel pretty worthless the way you are now?	Cognitive Distress	□Yes=1 □No=0
Do you feel full of energy?	Behavioral Activation	□Yes=0 □No=1
Do you feel that your situation is hopeless?	Cognitive Distress	□Yes=1 □No=0
Do you think that most people are better off than you are?	Cognitive Distress	□Yes=1 □No=0

**Total scores**		*A score > 5 points is suggestive of depression; ≥ 10 points is almost always indicative of depression; > 5 points should warrant a follow-up comprehensive assessment

Appendix 2. Athens Insomnia Scale (AIS)

<table>
<tr><th colspan="2">Question / Item</th><th>Score</th></tr>
<tr><td colspan="2">Sleep induction (time it takes you to fall asleep after turning-off the lights)</td><td>0=No problem
1=Slightly delayed
2=Markedly delayed
3=Very delayed or did not sleep at all</td></tr>
<tr><td colspan="2">Awakenings during the night</td><td>0=No problem
1=Minor problem
2=Considerable problem
3=Serious problem or did not sleep all</td></tr>
<tr><td colspan="2">Final awakening earlier than desired</td><td>0=Not earlier
1=A little earlier
2=Markedly earlier
3=Much earlier or did not sleep at all</td></tr>
<tr><td colspan="2">Total sleep duration</td><td>0=Sufficient
1=Slightly insufficient
2=Markedly insufficient
3=Very insufficient or did not sleep at all</td></tr>
<tr><td colspan="2">Overall quality of sleep (no matter how long you slept)</td><td>0=Satisfactory
1=Slightly unsatisfactory
2=Markedly unsatisfactory
3=Very unsatisfactory or did not sleep at all</td></tr>
<tr><td colspan="2">Sense of well-being during the day</td><td>0=Normal
1=Slightly decreased
2=Markedly decreased
3=Very decreased</td></tr>
<tr><td colspan="2">Functioning (physical and mental) during the day</td><td>0=Normal
1=Slightly decreased
2=Markedly decreased
3=Very decreased</td></tr>
<tr><td colspan="2">Sleepiness during the day</td><td>0=None
1=Mild
2=Considerable
3=Intense</td></tr>
<tr><td>Total scores</td><td></td><td>*The total score of AIS < 4 points is indicative of no sleep disorder; 4-6 points are suggestive as suspected insomnia; > 6 points indicate a diagnosis of insomnia.</td></tr>
</table>

Appendix 3. 3D-CAM

**Coding Instructions**: Incorrect also includes "I don't know", and No response/non-sensical responses. For any 'Incorrect' or 'Yes' responses, check the box in the final column designating which feature is present.		
**READ**: I have some questions about your thinking and memory.…		
1. Can you tell me the year we are in right now?	□Correct	□Incorrect
2. Can you tell me the day of the week?	□Correct	□Incorrect
3. Can you tell me what type of place is this? [hospital]	□Correct	□Incorrect
***If any of 3 items above are anything other than correct, feature 3 is present**		
4. I am going to read some numbers. I want you to repeat them in backwards order from the way I read them to you. For instance, if I say "5 – 2", you would say "2 -5". OK? The first one is "7-5-1" (1-5-7).	□Correct	□Incorrect
5. The second is "8-2-4-3" (3-4-2-8).	□Correct	□Incorrect
6. Can you tell me the days of the week backwards, starting with Saturday? [S,F,T,W,T,M,S] may prompt with "what is day before ….." for up to 2 prompts.	□Correct	□Incorrect
7. Can you tell me the months of the year backwards, starting with December? [D,N,O,S,A,J,J,M,A,M,F,J] may prompt with "what is month before ….." for up to 2 prompts.	□Correct	□Incorrect
***If any of items 4, 5, 6, or 7 above are anything other than correct, feature 2 is present**		
8. During the past day have you felt confused?	□No	□Yes
9. During the past day did you think that you were not really in the hospital?	□No	□Yes
10. During the past day did you see things that were not really there?	□No	□Yes
***If any of items 8, 9, or 10 above are anything other than 'no', feature 1 is present**		
Observer Ratings: To be completed after asking the patient questions 1-10 above		
11. Was the patient sleepy, stuporous, or comatose during the interview?	□No	□Yes
12. Did the patient show excessive absorption with ordinary objects in the environment (hypervigilant)?	□No	□Yes
***If either items 11A, 11B or 12 above are 'yes', feature 4 is present**		
13. Was the patient's flow of ideas unclear or illogical, for example tell a story unrelated to the interview (tangential)?	□No	□Yes
14. Was the patient's conversation rambling, for example did he/she give inappropriately verbose and off target responses?	□No	□Yes
15. Was the patient's speech unusually limited or sparse? (e.g. yes/no answers)	□No	□Yes
***If any of items 13, 14, or 15 above are 'yes', feature 3 is present**		
16. Did the patient have trouble keeping track of what was being said during the interview?	□No	□Yes

17. Did the patient appear inappropriately distracted by environmental stimuli?	□No	□Yes
***If either item 16 or 17 above are 'yes', feature 2 is present**		
18. Did the patient's level of consciousness fluctuate during the interview, for example, start to respond appropriately and then drift off?	□No	□Yes
19. Did the patient's level of attention fluctuate during the interview, e.g., did the patient's focus on the interview or performance on the attention tasks vary significantly?	□No	□Yes
20. Did the patient's speech/thinking fluctuate during the interview, for example, patient spoke slowly, then spoke very fast?	□No	□Yes
***If any of items 18, 19, or 20 above are 'yes', feature 1 is present**		
OPTIONAL QUESTIONS: COMPLETE ONLY IF FEATURE 1 IS NOT CHECKED AND FEATURE 2 IS CHECKED AND EITHER FEATURE 3 OR 4 IS CHECKED		
21. Contact a family member, friend, or health care provider who knows the patient well and ask: "Is there evidence of an acute change in mental status (memory or thinking) from the patient's baseline?"	□No	□Yes
22. IF SECOND DAY OF HOSPITALIZATION OR LATER AND PREVIOUS 3D-CAM RATINGS ARE AVAILABLE: Review previous 3D-CAM assessments and determine if there has been an acute change in performance, based on ANY new "positive" items	□No	□Yes

**CAM Summary: Check if Feature Present in column above**	**1**	**2**	**3**	**4**

DELIRIUM REQUIRES FEATURE 1 AND 2 and EITHER 3 OR 4:
______Present ______Not Present

Appendix 4. QoR-15 score

**READ**: How have you been feeling in the last 24 hours? (Have you had any of the following in the last 24 hours?)	
(0 to 10, where: 0 = none of the time [poor] and 10 = all of the time [excellent])	**Score**
1. Able to breathe easily	
2. Been able to enjoy food	
3. Feeling rested	
4. Have had a good sleep	
5. Able to look after personal toilet and hygiene unaided	
6. Able to communicate with family or friends	
7. Getting support from hospital doctors and nurses	
8. Able to return to work or usual home activities	
9. Feeling comfortable and in control	
10. Having a feeling of general well-being	
11. Moderate pain	
12. Severe pain	
13. Nausea or vomiting	
14. Feeling worried or anxious	
15. Feeling sad or depressed	
**Total Score**	
* QoR-15 > 135, excellent; 122 ≤ QoR-15 ≤ 135, good; 90 ≤ QoR-15 ≤ 121, moderate; QoR-15 < 90, poor. The higher the total score, the better the postoperative recovery.	

Appendix 5. Numeric rating scale (NRS)

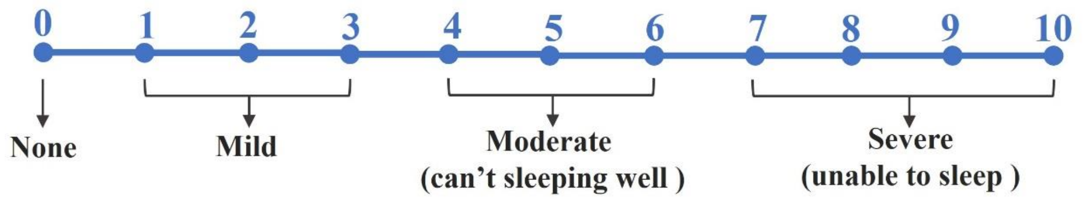

Supplement: Multimedia component 3 [file mmc3.pdf]
