# Supplementary material for: Intraoperative dexmedetomidine on postoperative sleep disturbance in older patients undergoing major abdominal surgery: A randomized controlled trial protocol
Source: Heliyon. 2024 May 21;10(11):e31668. doi: 10.1016/j.heliyon.2024.e31668 (PMC11153091; doi:10.1016/j.heliyon.2024.e31668)
Supplement: Multimedia component 4 [file mmc4.pdf]

苏州大学附属第一医院医学伦理委员会伦理审查批件

批件号：（2023）伦审批第 160 号

项目名称	右美托咪定对腹部大手术老年患者术后睡眠质量的影响：一项随机对照研究		
研究类别	临床科研项目		
申办方/发起方	苏州大学附属第一医院		
主要研究者	彭科	承担科室	麻醉科
伦理受理号	2023160	受理时间	2023-06-19
审查时间	2023-06-26	审查类别	初始审查
审查地点	总院综合楼 303 会议室	审查方式	会议审查
审查意见	依据我国相关法律、法规和国际伦理准则，伦理委员会对本项研究的研究方案、知情同意书、受试者招募材料及其他有关内容进行了会议审查，经投票表决，审查结果为同意开展本项研究。		
跟踪审查频率	本研究项目批准后 每 12 个月 向本伦理委员会递交研究进展报告。		

主任委员（签名）：陈罡

苏州大学附属第一医院医学伦理委员会（盖章）

日期：2023 年 06 月 27 日

研究者/申办方须知：

1. 请遵循 CFDA/GCP 和《赫尔辛基宣言》的原则、遵循伦理委员会批准的方案开展研究，保护受试者的健康和权利。研究者应严格按照所批准方案规定的期限和受试者例数完成研究，不得随意更改。
2. 对已批准的临床研究方案、知情同意书等材料的任何修改及主要研究者更换等，须再次提交伦理委员会重新审查，获得批准后方可执行。
3. 暂停/提前终止临床研究，请及时向伦理委员会报告。
4. 研究过程中发生需要上报的安全性事件，请按照要求向伦理委员会报告。
5. 发生违背方案的情况须及时向本伦理委员会报告。
6. 根据伦理委员会批件中规定的年度/定期跟踪审查频率，无论试验开始与否，请在持续审查日到期前 1 个月提出持续审查的申请并递交研究进展报告。
7. 完成临床研究，须及时提交结题报告供伦理委员会审查。
8. 本批件有效期一年（自批准之日起）。

扫描全能王 创建

审查文件清单
1. 临床研究方案（版本号：1.0，日期：2023-06-07）<br>2. 知情同意书（版本号：1.0，日期：2023-06-07）<br>3. 病例报告表（版本号：1.0，日期：2023-06-07）<br>4. 研究者简历<br>5. 研究者声明<br>6. 研究团队分工明细表<br>8. 利益冲突声明<br>9. 研究经费来源及研究成果发布形式说明<br>10. 涉及人的遗传资源使用管理声明

扫描全能王 创建

苏州大学附属第一医院医学伦理委员会
会议签到表

会议时间	2023 年 6 月 26 日（星期一）下午 13:30-16:30
会议地点	苏州大学附属第一医院总院综合楼 303 会议室

姓　名	性别	委员会任职	职　称	专　业	工作单位	签　名
陈　罡	男	主任委员	主任医师	神经外科	苏州大学附属第一医院	
朱雪松	男	副主任委员	研究员	基础医学	苏州大学附属第一医院	
徐溢涛	男	副主任委员	助理研究员	医院管理	苏州大学附属第一医院	
缪丽燕	女	委　员	主任药师	临床药学	苏州大学附属第一医院	
于树贵	男	委　员	教授	伦理学	苏州大学	
王进红	女	委　员	主任医师	内分泌科	苏州大学附属第一医院	
朱志伟	男	委　员	副教授	社会学	苏州大学	
张　华	男	委　员	副主任药师	临床药学	苏州大学附属第一医院	
陈　成	男	委　员	主任医师	呼吸科	苏州大学附属第一医院	
汤在祥	男	委　员	教授	卫生统计学	苏州大学	
陈苏宁	男	委　员	主任医师	血液科	苏州大学附属第一医院	
张拥军	男	委　员	律师	法学	北京隆安（苏州）律师事务所	
宋建平	女	委　员	主任医师	心血管内科	苏州大学附属第一医院	
唐晓文	女	委　员	主任医师	血液科	苏州大学附属第一医院	
章　斌	男	委　员	主任医师	核医学科	苏州大学附属第一医院	
韩　悦	女	委　员	主任医师	血液科	苏州大学附属第一医院	
魏雪栋	男	委　员	副主任医师	泌尿外科	苏州大学附属第一医院	

扫描全能王 创建
