# Supplementary material for: Intraoperative dexmedetomidine on postoperative sleep disturbance in older patients undergoing major abdominal surgery: A randomized controlled trial protocol
Source: Heliyon. 2024 May 21;10(11):e31668. doi: 10.1016/j.heliyon.2024.e31668 (PMC11153091; doi:10.1016/j.heliyon.2024.e31668)
Supplement: Multimedia component 5 [file mmc5.pdf]

受试者编号：　　　　　　　　　　　　　　　　　　　　　版本号：1.0

版本日期：2023 年 06 月 07 日

受试者知情同意书

（Informed Consent Form）

右美托咪定对腹部大手术老年患者术后睡眠质量的影响：一项随机对照研究

尊敬的女士 / 先生：

您将被邀请参加“右美托咪定对腹部大手术老年患者术后睡眠质量的影响：一项随机对照研究”的临床研究。下列各项记述了本研究的研究背景、目的、研究方法、研究过程中给您带来的益处和可能产生的不适、不便以及您的权益等，请您在参加临床研究前务必仔细阅读。本知情同意书提供给您一些信息以帮助您决定是否参加此项临床研究，如有任何疑问请向负责该项研究的医生提问，以确保您充分理解有关的内容。您是否参加本项研究是自愿的，假如您同意参加该临床研究，请您在知情同意书的签字页签字。

一、研究背景

术后睡眠障碍 (PSD) 作为术后常见症状之一，表现为睡眠剥夺、睡眠节律紊乱、睡眠体系异常、快慢波睡眠比例改变等，可导致认知功能下降、术后疼痛加剧、术后疲劳综合征及心血管意外事件的发生，最终影响术后恢复质量，减缓恢复速度。PSD 是多种因素共同作用的结果，包括手术炎症反应、疼痛、麻醉药物的使用、术后恶心呕吐以及对病房环境的不适应等。当前研究发现，右美托咪定有改善老年术后患者睡眠质量的潜力，但目前尚无研究明确不同剂量的右美托咪定对术后睡眠质量的改善程度是否有区别。

二、研究名称及目的

本研究的名称为：右美托咪定对腹部大手术老年患者术后睡眠质量的影响：一项随机对照研究。主要目的是：通过术中输注不同剂量的右美托咪定或安慰剂，评估对于腹部大手术老年患者术后 PSD 发生率、术后恢复质量、疼痛等情况的影响，为腹部大手术老年患者围术期麻醉用药管理提供实验依据，促进围术期恢复质量的改善。

三、研究方法和内容

本研究将由研究人员根据入组标准判断您是否适合参加。以 1:1:1 的比例将招募的参与者分配入组中，每组 70 例：（1）高剂量右美组、（2）低剂量右美组、（3）安慰剂组。为了在患者、围术期医疗团队和研究人员中实现对右美托咪定组的盲法，右美托咪定输注和安慰剂（0.9%生理盐水）以相等容积在同样的注射器中给药。通过收集您在常规临床麻醉以及术后恢复过程中产生的医疗信息数据资料，我们将对此医疗信息数据进行评价和分析。在术后 1-7 天、30 天我们将对您进行随访，并询问您关于术后恢复质量的相关问题并记录。在您了解整个研究的内容，您的问题都得到了满意的答复之后，如果您希望参加本次研究，您需签署这份知情同意书。研究者就开始安排进行相关的检查和研究操作。这些检查和研究操作将有助于确定您是否适合参加本次研究。这个阶段为“研究前阶段”。如果研究者判定您未达到入组标准，就不会允许您参加本次研究。研究者将会建议您按常规麻醉方案。

四、研究流程和时限

整个研究预计将在 1 年内完成。研究过程由筛选期间、住院期间、随访期间组成，您完成全部研究周期大约需要 1 个月，无需进行其他辅助检查。

五、参加研究可能的获益

您参与该项研究将免除相关药物（右美托咪定）的费用，同时有可能改善术后恢复质量，如减少术后睡眠障碍及术后谵妄发生的可能性。我们也希望通过研究所获得的信息了解到更多腹部大手术后镇痛的优选方案，为未来的老年患者的麻醉管理提供理论依据积累经验。

六、参加研究可能的风险和不适

本研究中的干预措施均在常规麻醉管理可接受范围内，因此，您参与此项研究，将不会给您增加超出常规诊疗以外的风险。可能存在的不良反应和治疗措施如下：术中如发生低血压（收缩压< 90 mmHg 或 MAP 降低幅度超过基础值 30%），则静脉注射麻黄素 10 mg；如发生严重心动过缓（HR< 45 次/min），则静脉注射阿托品 0.5 mg；若发生高血压（收缩压>140mmHg 或 MAP 升高幅度超过基础值 30%）和心动过速（HR>100 次/min），在麻醉深度和镇痛充分的情况下，可以静脉注射乌拉地尔 5 mg 或艾司洛尔 20 mg。若术中或术后发生低氧血症（SpO2＜90%），面罩加压辅助呼吸，行血气分析并对症处理。

如果有研究者以外的人获得了您的相关健康信息，可能会导致就业、保险或给家人带来麻烦。为了减少这些风险，我们将按相关规定保密您的个人信息。如一旦发生与研究相关的严重不良反应，采取相应的治疗措施，并且会立即通知伦理委员会，并按照中国的法律法规提供经济补偿。

七、受试者发生与研究相关伤害的治疗和经济补偿

受试者在研究过程中所出现的低血压、心动过缓、心动过速等上述不适，研究者应及时处理。如受试者发生与研究相关的伤害，研究发起方将根据我国相关法律法规承担相关的诊疗费用及相应的经济补偿。

八、本研究之外的常规诊疗方案

除参加本研究之外，您也可以选择采取不使用 BIS 监测、脑氧监测的常规麻醉管理。

九、受试者的权利

受试者参加研究享有包括自愿参加和随时退出、知情、同意或不同意、保密、受损害时获得免费治疗和补偿、在任何时候退出后不会遭到歧视或报复且医疗待遇与权益不会因此受到影响。

(1) 临床研究数据的保密方式

研究者负责遵循适用的数据保护条例来处理您的研究数据。但伦理委员会和上级行政管理部门视察能查阅到这些资料。研究结果可能全在医学刊物/会议上发表，但您的身份不会被告公开。

在签署该份知情同意书的后，即表明您同意研究医生及研究中心人员收集、使用和共享您的健康信息数据。在研究结束之前、研究结果得出之前，您允许我们使用您的健康信息的授权依然有效。但您可以随时通过研究负责医生撤回知情同意书。

十一、涉及人的生物样本的采集与管理

研究者声明本研究不涉及受试者的生物样本的采集与管理。

十二、联系方式

在您签这份同意书之前，研究组所有成员都会回答您的所有问题。如果您在签署该同意书之后，依然有问题、建议或意见，您还可以与研究者进行沟通。您可以随时了解本研究有

关的信息资料和研究进展。研究者及联系方式：彭科，15962155989，pengke@suda.edu.cn
伦理委员会联系人和联系方式：吴霜杰，0512-67972743

十三、声明与签署

受试者声明：我已经仔细阅读了本知情同意书，我有机会提问而且所有问题均已得到解答。我理解参加本项研究是自愿的，我可以选择不参加本项研究，或者在任何时候通知研究者后退出研究而不会遭到歧视或报复，我的任何医疗待遇与权益不会因此而受到影响。如果我需要其他治疗，或者我没有遵守研究计划，或者有任何其他合理的原因，研究医生可以终止我继续参与本项临床研究。我自愿同意参加该临床研究，我将收到一份签过字的“知情同意书”副本。

受试者姓名（正楷）________________；签名：________________；

日期：______年___月___日；手机号码：_________________。

法定代理人姓名（正楷）________________；法定代理人签名：_________________；

日期：________年____月____日；手机号码：__________________；与受试者的关系：__________；

受试者不能签署知情同意书的理由：____。

研究者声明：我已准确地将知情同意书内容告知受试者并对受试者的提问进行了解答，受试者自愿参加本项临床研究。

研究者姓名（正楷）________________；研究者签名：_________________；

日期：______年___月___日；手机号码：
